# Do Girls Have a Nutritional Disadvantage Compared with Boys? Statistical Models of Breastfeeding and Food Consumption Inequalities among Indian Siblings

**DOI:** 10.1371/journal.pone.0107172

**Published:** 2014-09-17

**Authors:** Jasmine Fledderjohann, Sutapa Agrawal, Sukumar Vellakkal, Sanjay Basu, Oona Campbell, Pat Doyle, Shah Ebrahim, David Stuckler

**Affiliations:** 1 Department of Sociology, Oxford University, Oxford, United Kingdom; 2 South Asia Chronic Disease Network, Public Health Foundation of India, New Delhi, India; 3 Stanford Prevention Research Center, Stanford University School of Medicine, Stanford, CA, United States of America; 4 Department of Non-Communicable Disease Epidemiology, London School of Hygiene & Tropical Medicine, London, United Kingdom; Radboud University, Netherlands

## Abstract

**Background:**

India is the only nation where girls have greater risks of under-5 mortality than boys. We test whether female disadvantage in breastfeeding and food allocation accounts for gender disparities in mortality.

**Methods and Findings:**

Secondary, publicly available anonymized and de-identified data were used; no ethics committee review was required. Multivariate regression and Cox models were performed using Round 3 of India’s National Family and Health Survey (2005–2006; response rate = 93.5%). Models were disaggregated by birth order and sibling gender, and adjusted for maternal age, education, and fixed effects, urban residence, household deprivation, and other sociodemographics. Mothers’ reported practices of WHO/UNICEF recommendations for breastfeeding initiation, exclusivity, and total duration (ages 0–59 months), children’s consumption of 24 food items (6–59 months), and child survival (0–59 months) were examined for first- and secondborns (n = 20,395). Girls were breastfed on average for 0.45 months less than boys (95% CI: = 0.15 months to 0.75 months, p = 0.004). There were no gender differences in breastfeeding initiation (OR = 1.04, 95% CI: 0.97 to 1.12) or exclusivity (OR = 1.06, 95% CI: 0.99 to 1.14). Differences in breastfeeding cessation emerged between 12 and 36 months in secondborn females. Compared with boys, girls had lower consumption of fresh milk by 14% (95% CI: 79% to 94%, p = 0.001) and breast milk by 21% (95% CI: 70% to 90%, p<0.000). Each additional month of breastfeeding was associated with a 24% lower risk of mortality (OR = 0.76, 95% CI: 0.73 to 0.79, p<0.000). Girls’ shorter breastfeeding duration accounted for an 11% increased probability of dying before age 5, accounting for about 50% of their survival disadvantage compared with other low-income countries.

**Conclusions:**

Indian girls are breastfed for shorter periods than boys and consume less milk. Future research should investigate the role of additional factors driving India’s female survival disadvantage.

## Introduction

India is the only country in the world where young girls have worse under-five mortality than boys. In 2012, the under-5 mortality ratio in India was estimated at 108.5 female deaths for every 100 male deaths [1], [2]. Although Indian girls do have better neonatal mortality outcomes, female infant mortality in the postneonatal period outweighs this period of advantage. Over the past four decades, declines in infant mortality have been greater in boys than girls, further widening inequalities in India’s male-female child survival rates [3]. This trend starkly contrasts with the rest of the world, where young girls have clear survival advantages [4]. For example, in neighbouring Bangladesh, the sex mortality ratio is 86.4 female per 100 male deaths [5], [6]. Globally, this ratio stands at 92.5 female per 100 male deaths, and in the UN’s Least Developed Countries, girls tend to fare even better, averaging 88.3 female per 100 male deaths [5], [6].

It is unclear why young girls fare worse than boys in India. While extensive research has documented son preference in India [7]–[9], establishing that sex-selective abortion and female infanticide leads to smaller numbers of girls [10], the reasons for lower infant and child survival is not clear [11]–[13]. One leading hypothesis is that son preference continues into childhood, with families giving boys more food than girls. India has a high burden of malnutrition [14]; 60 million children are underweight, and child malnutrition alone accounts for 22% of the disease burden in India [15]. However, findings on gender disparities in malnutrition from national surveys provide ambiguous evidence, including some evidence of a male disadvantage in stunting and wasting. At a population level, these studies have been critiqued as a potential artefact of lower survival rates of the most malnourished females [16].

There is a dearth of recent studies evaluating potential nutritional bias against young girls in India. Studies from the Philippines [17] and Sri Lanka [18] indicate that maternal education influences duration and quality of breastfeeding, but have not tested the possibility of gender differentials. Analyses of National Rural Household surveys from India in the 1970s and 1980s reported mixed results, with some finding, variously, that females have better nutrition outcomes than males [19], worse outcomes [20], [21], or no sex-related difference [22], [23]. An important limitation was that these studies drew on data primarily from rural villages, overlooking urban differences [20], [24], and relied on anthropometric measures as proxies for nutritional intake, which may be confounded by female mortality patterns.

One analysis using India’s National Family and Health Survey (NFHS) from 1992–93 found that the first girl in the family fares better on immunization and anthropometry than boys with many older brothers, while girls with many sisters are the most disadvantaged group as a result of a desire for a balanced sex composition of children [25]. Another NFHS analysis of children under 36 months found evidence of sex-selective disadvantage in the duration of breastfeeding depending on birth order and desired family size [26]. When mothers desired additional children than the current number, both females and males appeared to experience disadvantage. While these studies suggest that young females may receive less food, their scope has been limited to isolated factors, such as breastfeeding duration, and, to our knowledge, have not investigated food intake more generally. Previous work has also yet to investigate potential gender disparities in WHO and UNICEF recommended practices to initiate breastfeeding within the first hour of life, exclusively breastfeed for the first 6 months, and introduce solid foods at 6 months [27].

To test whether Indian girls are disadvantaged in WHO/UNICEF recommended feeding practices, we evaluated the individual and household data covering breastfeeding, nutrition, and child health for 20,395 siblings ages 0–59 months from the latest available round of India’s NFHS. We hypothesised that young girls would have a lower duration of breastfeeding, particularly when the firstborn child was female, as well as lower access to more expensive, high-protein foods such as meat and fish. As a further test we evaluated whether feeding inequalities could account young girls’ survival disadvantage in India relative to boys.

## Methods

### Source of Data

Data for the study were completely anonymized and de-identified prior to access, and were obtained from a third party (USAID). No research ethics committee review was required. All data are publicly available by request to DHS [28]. Data on 14,801 Indian mothers and their 20,395 children were taken from the nationally representative National Family and Health Surveys (NFHS-3; 28). 19,305 (95%) of these children were alive at the time of interview. The full sample of the NFHS-3 includes data on 124, 385 women aged 15–49, and includes information on reproductive histories, breastfeeding practices, frequency food item consumption for mothers and children, as well as anthropometric measures. Child nutrition data on breastfeeding and food item consumption were available for children born in the 5 years preceding the survey. To compare children of equal birth order and sibling composition, we restricted the sample to women whose first two children were born in the five years from 2000–2005. To evaluate potential gender bias in breastfeeding, food intake, and nutritional outcomes, we compared male and female children for firstborn and secondborn children separately. Thirdborns were not disaggregated due to small numbers (n = 719). Missing data were handled using listwise deletion, in which cases missing on any variable in the analysis are excluded; missingness was <7% for all variables included in the analyses except where child mortality resulted in legitimate missing, such as reports of food consumption for living children.

Table 1 provides descriptive statistics for the sample, split by gender. In this sample, a higher proportion of females were alive than males, reflecting the period rather than cohort construction of the sample. To age-adjust these figures, we applied Cox-survival models, described further below, which shows that age-adjusted hazard rates were 1.17 (CI: 0.90 to 1.51; p = 0.24) in females and 0.86 (CI: 0.66 to 1.11; p = 0.24) in males.

**Table 1:** Descriptive statistics for all children, NFHS-3.

	Females			Males		
	N	Mean/%	St. Dev	N	Mean/%	St. Dev
Child’s Age (Months)		25.2	(17.22)		24.9	(17.09)
Mother’s Age (Years)		23.9	(4.15)		23.9	(4.10)
Place of residence						
Urban	4328	43.7%	(0.50)	4628	44.1%	(0.50)
Rural	5572	56.3%	(0.50)	5867	55.9%	(0.50)
Mother’s Education (Highest Level Completed)						
No Schooling	2580	26.1%	(0.44)	2756	26.3%	(0.44)
Primary School	1381	14.0%	(0.35)	1467	14.0%	(0.35)
Secondary School	4820	48.7%	(0.50)	5040	48.0%	(0.50)
Higher than Secondary	1118	11.3%	(0.32)	1232	11.7%	(0.32)
Deprived Household	2700	27.3%	(0.45)	2678	25.5%	(0.44)
Duration of Breastfeeding (Months)		14.5	(10.09)		14.9	(10.11)
Breastfed within 1 Hour of Birth	4548	46.3%	(0.50)	4661	44.6%	(0.50)
Breastfed Exclusively for 6 Months	4007	49.7%	(0.50)	4343	50.0%	(0.50)
Breastfed for 6+ Months	8805	89.8%	(0.30)	9290	89.5%	(0.31)
Index of Child’s Food Consumption Last 24 Hours	8206	6.19	(4.39)		6.23	(4.31)
Consumed Last 24 Hours: Nothing	360	4.44%	(0.21)	329	3.88%	(0.19)
Consumed Last 24 Hours: Water	6925	84.4%	(0.36)	7281	85.1%	(0.36)
Consumed Last 24 Hours: Juice	960	11.7%	(0.32)	1022	12.0%	(0.32)
Consumed Last 24 Hours: Tea/Coffee	2857	34.9%	(0.48)	3066	35.9%	(0.48)
Consumed Last 24 Hours: Powdered/Fresh Milk	3832	46.7%	(0.50)	4178	48.8%	(0.50)
Consumed Last 24 Hours: Formula	1020	12.5%	(0.33)	1085	12.7%	(0.33)
Consumed Last 24 Hours: Baby Cereal	1440	17.6%	(0.38)	1452	17.0%	(0.38)
Consumed Last 24 Hours: Porridge/Gruel	1822	22.2%	(0.42)	1860	21.7%	(0.41)
Consumed Last 24 Hours: Other Liquids	1324	16.2%	(0.37)	1461	17.1%	(0.38)
Consumed Last 24 Hours: Chicken, Duck, Other Birds	186	2.27%	(0.15)	185	2.20%	(0.15)
Consumed Last 24 Hours: Meat	260	3.18%	(0.18)	252	3.00%	(0.17)
Consumed Last 24 Hours: Beans, Peas, or Lentils	985	12.0%	(0.33)	956	11.2%	(0.32)
Consumed Last 24 Hours: Nuts	609	7.44%	(0.26)	607	7.10%	(0.26)
Consumed Last 24 Hours: Bread, Noodles, Other Grains	5034	61.4%	(0.49)	5386	62.9%	(0.48)
Consumed Last 24 Hours: Potatoes, Cassava, Tubers	1594	19.4%	(0.40)	1695	19.8%	(0.40)
Consumed Last 24 Hours: Eggs	713	8.72%	(0.28)	754	8.80%	(0.28)
Consumed Last 24 Hours: Pumpkin, Carrots, Squash	1023	12.5%	(0.33)	1109	13.0%	(0.34)
Consumed Last 24 Hours: Dark Green Leafy Vegetables	1946	23.8%	(0.43)	1992	23.3%	(0.42)
Consumed Last 24 Hours: Mangoes, Papayas, Vitamin A Fruits	1135	13.8%	(0.35)	1194	14.0%	(0.35)
Consumed Last 24 Hours: Other Fruits/Vegetables	1543	18.8%	(0.39)	1668	19.5%	(0.40)
Consumed Last 24 Hours: Liver, Heart, Other Organ Meat	191	2.33%	(0.15)	204	2.40%	(0.15)
Consumed Last 24 Hours: Fresh/Dried Fish, Shellfish	595	7.27%	(0.26)	610	7.10%	(0.26)
Consumed Last 24 Hours: Food from Beans, Peas, Lentils, Nuts	1350	16.5%	(0.37)	1333	15.6%	(0.36)
Consumed Last 24 Hours: Cheese, Yogurt, Milk Products	1076	13.1%	(0.34)	1118	13.1%	(0.34)
Consumed Last 24 Hours: Oil, Fats, Butter	1160	14.2%	(0.35)	1202	14.1%	(0.35)
Consumed Last 24 Hours: Other Solid/Semi-Solid Food	1447	17.7%	(0.38)	1451	17.0%	(0.38)
Consumed Last 24 Hours: Any Protein (Excluding Breast milk)	5090	62.0%	(0.49)	5490	64.2%	(0.48)
Consumed Last 24 Hours: Any Protein (Including Breast milk)	7070	84.3%	(0.36)	7528	86.0%	(0.35)
Consumed Last 24 Hours: Any Vitamin A Fruits/Vegetables	2897	35.3%	(0.48)	3062	35.8%	(0.48)
Dietary Diversity Scale	8175	2.34	(1.86)		2.36	(1.84)
Child is Alive	9416	95.1%	(0.22)	9889	94.2%	(0.23)

### Breastfeeding measures

Breastfeeding outcomes included three measures of compliance with WHO and UNICEF recommended breastfeeding practices, including the initiation of breastfeeding (which should be within one hour of birth), duration of breastfeeding (which should be exclusive for the first 6 months), and the introduction of complementary solid, semi-solid, and soft foods at six months [27]. We measured exclusive breastfeeding based on the mother’s response to a series of questions asking what food and liquid items children were given in the first three days of life. Children aged 6–59 months whose breastfeeding duration was less than six months and those receiving anything other than breast milk, including water, in the first three days of life were coded as non-exclusive; children who received only breast milk in the first three days and whose breastfeeding duration was at least six months were coded as exclusively breastfeeding. Total breastfeeding duration was based on mother’s report of child’s age (in months) at breastfeeding cessation among children who had initiated breastfeeding.

### Food intake measures

Food intake was measured using the mother’s report of whether and how frequently the child received milk, eggs, fruit and vegetables, and other specific food items in the 24 hour period preceding the interview; a food frequency index (summing the mother’s yes/no food item report, with additional weighting for high-protein sources); and a dietary diversity index [30]. Data on breastfeeding were collected for children who were no longer living at the time of the survey. These children were included in breastfeeding analyses, but were excluded from analyses of current nutritional intake, as food consumption variables were unavailable for non-surviving children. Child survival was assessed from mother’s reports of whether the child was alive at the time of the survey.

### Statistical Models

We used multivariate fixed-effects linear and logistic regression models to assess gender disparities in feeding practices. Models were adjusted for maternal age, a household asset index, and other potential confounding factors as follows:
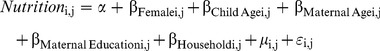
(1)


Here *i* is the child and *j* is the mother; nutrition includes measures of whether the child was breastfed within an hour of birth, exclusivity and duration of breastfeeding, food item consumption, and dietary diversity; female is the child’s gender (1 = female, 0 = male); child age is in months; maternal age is in years, and education is a categorical measure of mother’s highest level of education (no schooling, primary school, secondary school, or higher than secondary). 98.1% of mothers were married, so marital status was not included due to small numbers of unmarried mothers. Household is a vector of household characteristics, including a dummy variable for economic deprivation based on the standard DHS index of household wealth (lowest, lower wealth = deprived; middle, higher, highest wealth = not deprived) and place of residence (rural or urban) [29]. µ is a mother fixed effect, and ε is the error term.

Breastfeeding duration was evaluated using Kaplan Meier survival curves. Following previous studies [26], we dropped children who died (n = 1,134), which resulted in the exclusion of about 5.4% of the sample. However, in a series of robustness checks, we found that including these children did not alter the findings qualitatively (survival probabilities differed by <0.0002). Models disaggregated by birth order are presented as marginal probabilities. To account for the large number of comparisons across individual food items, standard errors were adjusted using a Bonferroni correction. Cox survival models were used to assess the hazard of dying associated with breastfeeding practices. Population weights were applied in all analyses to account for the sampling design of the NFHS [29]. All analyses were conducted using Stata version 12.1.

## Results

### Testing female disadvantage in WHO breastfeeding practices

Table 2 provides the results of multivariate logistic regression models of the odds of adhering to WHO best practices regarding initiation, exclusivity, and duration of breastfeeding (6+ months), and of overall duration of breastfeeding comparing boys and girls. After correcting for maternal age, education, household deprivation and other sociodemographic confounders, we found that girls faced a significant disadvantage in duration of breastfeeding. Young girls, on average, were breastfed for 0.45 fewer months than boys (95% CI: −0.76 to −0.13). However, no gender differences were observed with regard to other WHO best breastfeeding practices, including the likelihood of being put to breast within one hour of birth (OR = 1.04, 95% CI: 0.97 to 1.12), exclusive breastfeeding for the first six months of life (OR = 1.02, 95% CI: 0.94 to 1.10), and breastfeeding duration of at least six months (OR = 1.07, 95% CI: 0.95 to 1.22).

**Table 2:** WHO best breastfeeding practices, duration (in months), and gender, children aged 6–59 months, NFHS-3.

		*Put to Breast within 1 Hour*		*Exclusively Breastfed First 6 Months*		*Breastfed 6+ Months*		*Breastfeeding Duration* †	
		OR	95% CI	OR	95% CI	OR	95% CI	B	95% CI
Female		1.04	[0.97 to 1.12]	1.02	[0.94 to 1.10]	1.07	[0.95 to 1.22]	−0.45**	[−0.75 to −0.15]
Child’s Age (Months)		1.00***	[1.00 to 1.01]	1.02***	[1.02 to 1.02]	1.01**	[1.00 to 1.01]	0.33***	[0.33 to 0.35]
Mother’s Age (Years)		1.03***	[1.02 to 1.04]	1.00	[0.99 to 1.01]	0.96***	[0.94 to 0.98]	0.00	[−0.06 to 0.03]
Urban Residence		1.03	[0.94 to 1.12]	0.92	[0.83 to 1.01]	0.80**	[0.70 to 0.92]	−1.12***	[−1.46 to −0.76]
Mother’s Education									
No Schooling (Ref)									
Primary School		1.68***	[1.49 to 1.89]	1.41***	[1.24 to 1.61]	1.04	[0.85 to 1.27]	0.02	[−0.48 to 0.48]
Secondary School		2.08***	[1.89 to 2.30]	1.60***	[1.43 to 1.77]	1.11	[0.95 to 1.31]	0.04	[−0.31 to 0.48]
Higher than Secondary		1.83***	[1.56 to 2.15]	1.76***	[1.48 to 2.09]	1.21	[0.95 to 1.54]	−0.92**	[−1.43 to −0.18]
Deprived Household		1.08	[0.98 to 1.19]	1.05	[0.95 to 1.17]	1.16	[0.99 to 1.37]	0.82***	[0.36 to 1.12]
Mother’s ID (Fixed Effects)		1.00***	[1.00 to 1.00]	1.00***	[1.00 to 1.00]	1.00	[1.00 to 1.00]	0.00	[−0.00 to 0.00]
Number of Children		20085		16624		19999		19004	
(Pseudo) R-Squared		0.03		0.03		0.01		0.31	

Notes: *p<.05 **p<.01 ***p<.001.

† Linear regression model.

To test whether young girls were more disadvantaged in the duration of breastfeeding when the prior siblings were also girls, we disaggregated our results by birth order and sibling gender. Among firstborns, there was no gender disparity (B = −0.32, 95% CI: −0.71 to 0.08). However, when the older sibling was female, secondborn females had 0.85 months shorter duration of breastfeeding than males (95% CI: −1.45 to −0.25). In contrast, no significant gender disparity in breastfeeding duration was observed when the first sibling was male (B = 0.34, 95% CI: −0.92 to 0.24).

**Figure 1:**
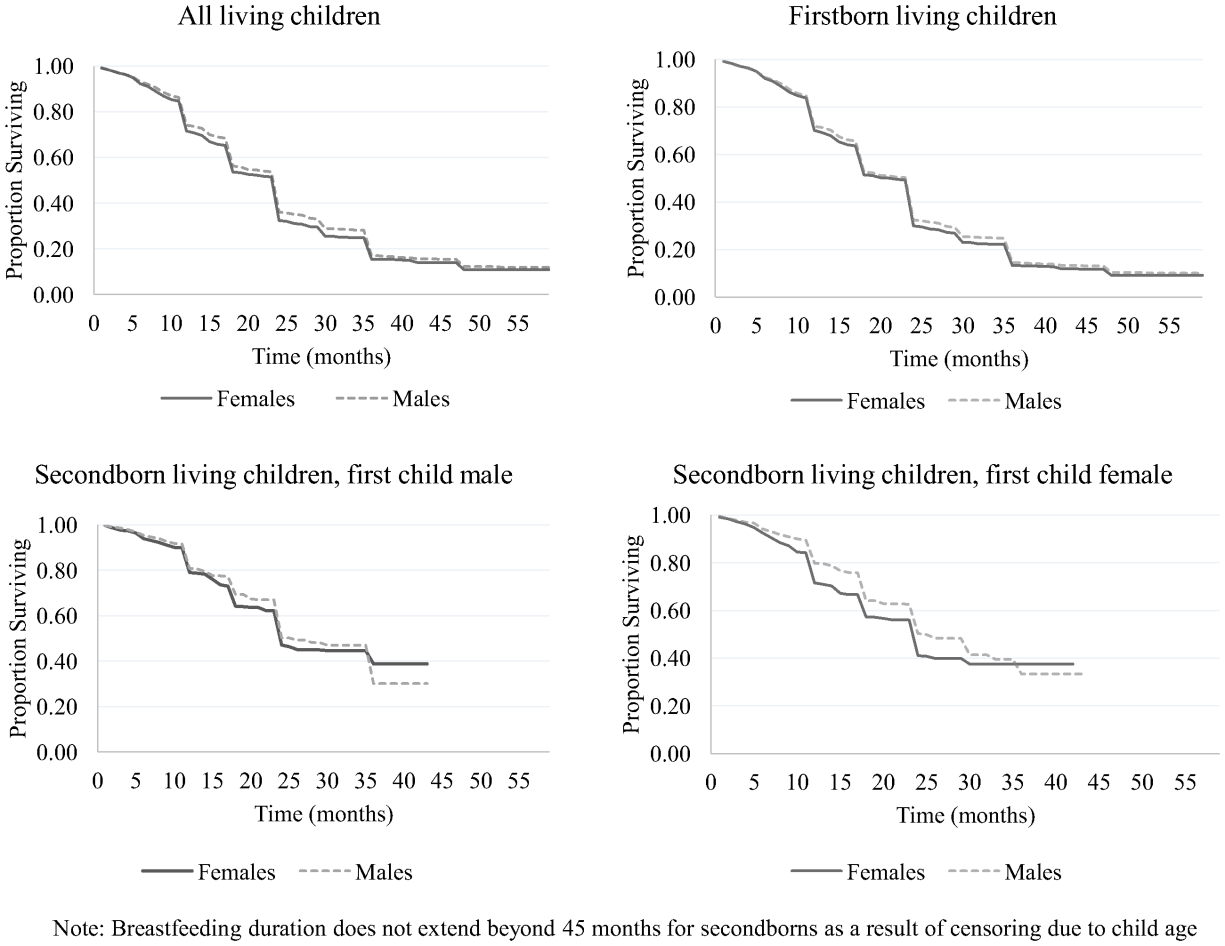
Kaplan Meier curves for breastfeeding duration by gender and birth order, NFHS-3.

Figure 1 depicts the Kaplan Meier survival curves for overall breastfeeding duration by sex. As shown in the figure, breastfeeding patterns are similar for boys and girls until about 12 months of age, when a gender gap begins to emerge. Median time to cessation of breastfeeding was 23.2 months for both girls and boys. Among firstborns, median duration of breastfeeding was around 21.0 months for females and 23.2 months for males, indicating earlier weaning of firstborn females. Irrespective of whether the firstborn child was female or male, secondborn females experienced a slight disadvantage in median breastfeeding survival duration (23.1 months for females and 24.0 for males).

### Testing female disadvantage in food consumption

Table 3 tests whether girls aged 6–59 months were given the same food items as boys in the last 24 hours, with p-values adjusted using a Bonferroni correction. Girls were significantly less likely to have consumed fresh milk (OR = 0.86, 95% CI: 0.78 to 0.94) and breast milk (OR = 0.79, 95% CI: 0.70 to 0.90). No gender disparities were found for the other food items, including high-protein foods such as poultry, fish, and meat. Nor was there evidence of a significant gender difference in dietary diversity.

**Table 3:** Odds of specific food item consumption for females compared with males, living children aged 6–59 months, NFHS-3.

Food Item	OR	95% CI
Milk	0.86**	[0.78 to 0.94]
Baby Formula	1.05	[0.92 to 1.21]
Breast milk	0.79***	[0.70 to 0.90]
Chicken, Duck, or Other Birds	0.92	[0.66 to 1.27]
Any Other Meat	1.09	[0.82 to 1.44]
Beans, Peas, or Lentils	1.01	[0.88 to 1.16]
Nuts	1.10	[0.94 to 1.29]
Eggs	0.96	[0.81 to 1.12]
Liver, Heart, or Other Organs	0.93	[0.67 to 1.28]
Fish/Shellfish	1.15	[0.97 to 1.38]
Food from Beans, Peas, Lentils, or Nuts	1.05	[0.93 to 1.18]
Cheese, Yogurt, Dairy	1.02	[0.90 to 1.16]
Baby Cereal	1.08	[0.96 to 1.22]
Gruel/Porridge	0.99	[0.89 to 1.10]
Bread, Noodles, or Other Grains	0.87	[0.79 to 0.96]
Potatoes, Cassava, or Other Tubers	0.97	[0.87 to 1.07]
Pumpkin, Carrots, Squash	0.99	[0.87 to 1.12]
Dark Green Leafy Vegetables	1.02	[0.92 to 1.13]
Mangoes, Papayas, Vitamin A Fruit	0.98	[0.87 to 1.12]
Other Fruits/Vegetables	0.97	[0.87 to 1.08]
Oil, Fats, Butter	0.96	[0.85 to 1.09]
Other Solid/Semi-Solid Food	1.00	[0.90 to 1.12]

*Notes:* Sample size 14,821 children. All models were adjusted for child’s age in months, maternal age and a categorical measure of educational attainment, household deprivation, and urban/rural residence, as well as mother fixed effects. Standard errors include Bonferroni correction to adjust for multiple comparisons.

*p<.05 **p<.01 ***p<.001.

**Figure 2:**
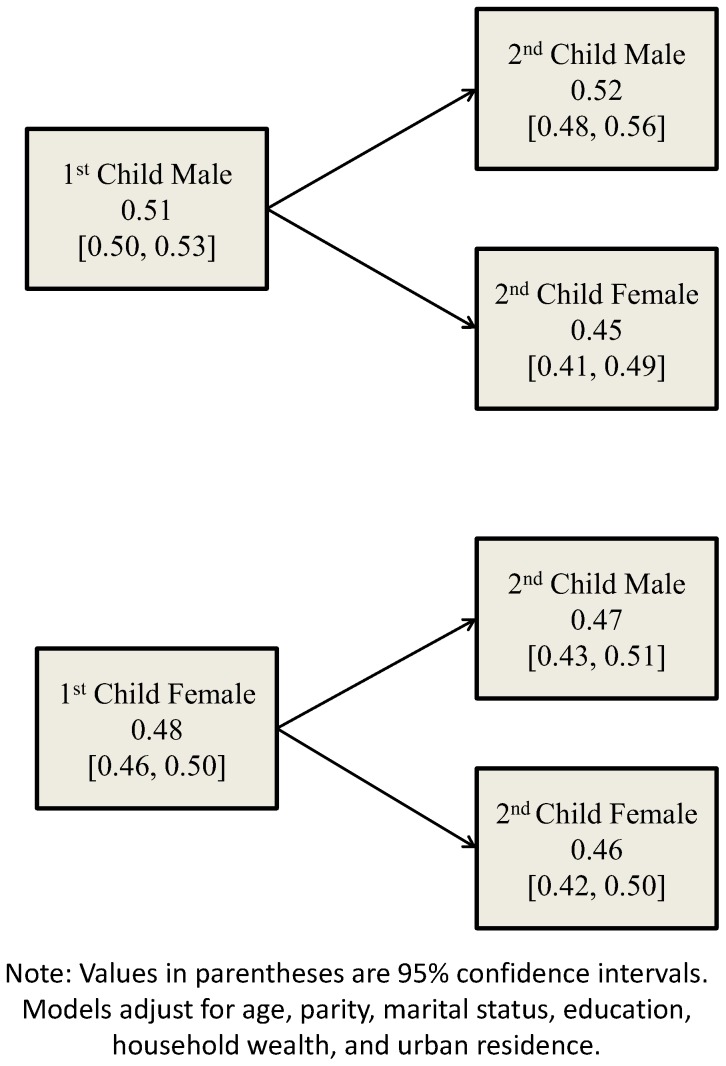
Predicted probabilities of receiving fresh milk, children <60 months, NFHS-3.

**Figure 3:**
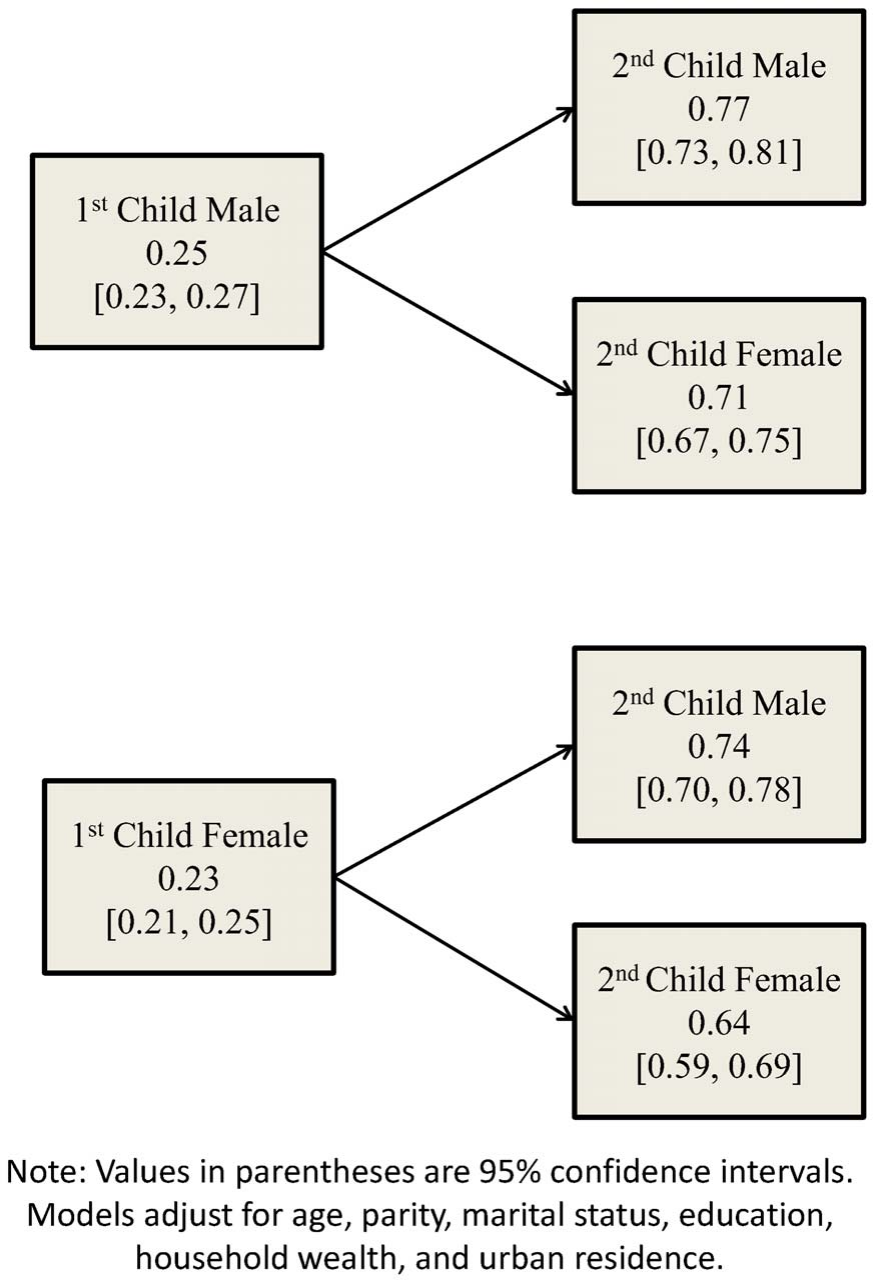
Predicted probabilities of receiving breast milk, children <60 months, NFHS-3.

Figure 2 shows the predicted probabilities of fresh milk consumption by birth order and gender. Firstborn girls have a slightly lower probability of consuming fresh milk than firstborn boys. However, while there is only a negligible difference in the probabilities for boys and girls when the first child was female (0.47 for males versus 0.46 for females), males with an older brother have a much higher probability of consuming fresh milk compared to girls with an older brother (0.52 versus 0.45). A replication of these disaggregated models for breast milk is depicted in Figure 3. As shown, overall probabilities across sex are similar among firstborns, but a gender discrepancy emerges among secondborns. Secondborn girls have a lower probability of consuming breast milk than boys irrespective of whether their older sibling was female (0.64 for girls versus 0.74 for boys) or male (0.74 for girls versus 0.77 for boys). Girls with an older sister were observed to have the lowest probability of consuming breast milk, while boys with an older brother had the highest probability.

### Robustness Tests

We tested the robustness of our models to alternative specifications. As children who die would experience a shorter duration of breastfeeding, we re-estimated models including children who died. Results were unchanged. Since mothers who have had a caesarean section are less likely to breastfeed within the first hour of birth as they are in recovery from surgery, we included delivery by caesarean as a robustness check in the model estimating odds of breastfeeding within one hour of birth. There was no change to our basic findings. Although we included mothers’ fixed effects, it is possible that there was unobserved heterogeneity by Indian state. We added 28 dummy variables for each Indian state, finding none of our main results was changed. Finally, it was possible that religious affiliation (Hindu, Muslim, Christian, or other) and/or caste (schedule caste, schedule tribe, none, other) affected the results. When we included these covariates, we still observed that young girls received a lower duration of breastfeeding than boys.

### Association of Female Breastfeeding Disadvantage with Gender Disparities in Child Survival

To assess the clinical significance of the gender disparities, we evaluated the hazard of dying in association with breastfeeding practices using Cox survival models, as shown in Table 4. Consistent with previous findings, we observed that longer duration of breastfeeding was associated with a lower risk of mortality (HR = 0.76, 95% CI: 0.73 to 0.79), after adjusting for educational attainment, household deprivation, and other maternal characteristics and sociodemographic confounders.

**Table 4:** Hazard of mortality and duration (in months) of breastfeeding by gender, children aged 0–59 months, NFHS-3.

	*All Children*		*Females*		*Males*	
	HR	95% CI	HR	95% CI	HR	95% CI
Duration of Breastfeeding	0.76***	[0.73 to 0.79]	0.74***	[0.70 to 0.78]	0.78***	[0.74 to 0.82]
Mother’s Age (Years)	0.96	[0.92 to 1.00]	0.93*	[0.88 to 0.99]	0.98	[0.92 to 1.05]
Urban Residence	0.60**	[0.41 to 0.87]	0.60	[0.35 to 1.02]	0.57*	[0.34 to 0.96]
Mother’s Education						
No Schooling (Ref)						
Primary School	0.62*	[0.42 to 0.90]	0.45**	[0.25 to 0.80]	0.83	[0.50 to 1.37]
Secondary School	0.37***	[0.26 to 0.53]	0.32***	[0.20 to 0.53]	0.41**	[0.24 to 0.70]
Higher than Secondary	0.27**	[0.11 to 0.69]	0.13**	[0.03 to 0.59]	0.45	[0.14 to 1.49]
Deprived Household	1.53*	[1.10 to 2.13]	1.21	[0.75 to 1.95]	1.90**	[1.21 to 3.00]
Number of Children	19259		9406		9853	
Pseudo R-Squared	0.14		0.17		0.13	

*Notes:* *p<.05 **p<.01 ***p<.001.

There was no statistically distinguishable heterogeneity by sex when splitting the sample by gender. As an alternative specification (not shown), we performed a mediation analysis by first modelling mortality as a function of gender, maternal and sociodemographic characteristics, excluding the effect of breastfeeding, then adding breastfeeding to the model. The model that did not control for the effect of breastfeeding identified a hazard rate of 1.17 (CI: 0.88 to 1.52; p = 0.24), while the addition of breastfeeding to the model resulted in a reduced but still non-significant gender coefficient (HR = 1.08; CI: 0.82 to 1.42). All other coefficients were in the expected direction, including protective associations for higher education and adverse ones for household deprivation. As a robustness check, we re-estimated the Cox proportional hazards models including two measures of birth weight: mothers’ assessment of the child’s relative size and the birth weight in kilograms recorded from health cards (only available for 10% of the sample). Neither changed the estimated effect size of breastfeeding duration on increased survival, although in the case of the latter the confidence intervals widened due to smaller sample size.

In order to gauge the portion of the gender gap in the hazard of dying explained by breastfeeding duration, we multiplied the reduction in the hazard of dying for every month increase in breastfeeding duration (0.24) given by 1.00 minus the breastfeeding duration coefficient in Table 4 by the overall gender gap in breastfeeding duration (0.45 months) from Table 2. This yields an estimate that breastfeeding duration explains only 10.8% of the gender gap in the hazard of dying, which corresponds to about one-half of the 20.2% gender gap in survival in India as compared with other high-poverty nations.

## Discussion

Our study evaluates whether young girls’ lower survival than boys in India relates to differential adherence to WHO and UNICEF recommended nutritional practices. We compared breastfeeding initiation, exclusivity, and duration among siblings. Our results demonstrate that, on average, Indian girls experience about one-half month shorter breastfeeding duration than boys. This risk was concentrated in secondborn girls and appeared to emerge after the first 12 months of life. Girls were also less likely to have received breast milk and fresh milk as a source of protein in the last 24 hours compared with boys. There were no statistically detectable gender differentials in access to lentils, meat, fish, and other high-protein foods.

While previous studies have found that duration of breastfeeding may be a significant predictor of child survival [31], [32], the magnitude of findings here suggests that gender differences are likely to account for a significant proportion of young girls’ disproportionately worse survival outcomes relative to boys in India compared with the rest of the world. Consistent with earlier epidemiological studies, which found each added month of breastfeeding reduced infant deaths by 6 per 1,000 live births [33], our study confirmed that longer duration of breastfeeding confers significant survival benefits, with each month reducing risk of dying by 24% (approximately similar in effect size). The magnitude of this protective effect, combined with the observed gender gap of about 0.45 months, meant that it could explain an additional 11% risk of dying in young girls between ages 0 and 5, corresponding to approximately half of the gap in male-female survival ratios between India and other developing countries.

Our study has several important limitations. First, data on the quantity of food in different categories were unavailable. Thus, it is possible that boys and girl receive food with equal frequency, but in differing quantities. Second, data on food intake relies on mothers’ reports. This may lead to measurement error if mothers underreport disparities in feeding practices. However, this would conservatively bias findings, tending to understate the magnitude of true differences between boys’ and girls’ nutrition. Third, it is possible that mothers may not report differential access to breastfeeding and food (a social desirability bias). However, anthropometric data are consistent with the observed breastfeeding and nutritional practices in the study. Fourth, due to sample size limitations, our analysis was limited to first and second births; however, gendered mortality risk may be higher among children of higher birth order [34], [35]. Thus, we may understate the full effect of gendered breastfeeding patterns on mortality. Finally, our measure of exclusivity of breastfeeding is based on reported feeding practices during the first 3 days of life and the preceding 24 hour period. As a result, we were unable to measure the precise timing of when solid foods were introduced to the diet. It is likely that there are residual gender differences in exclusive breastfeeding beyond 6 months, and that extending the duration of exclusive breastfeeding could have detrimental effects on infants’ growth and health [27].

Turning to our hypotheses, we found partial evidence for the hypothesis that young girls are less likely to be fed according to WHO/UNICEF recommended breastfeeding practices. We did not find evidence to support the claim that young girls would be disadvantaged compared to boys because mothers were weaning their first- or secondborn daughters in hopes of giving birth to son. Although breastfeeding duration was lower in secondborn children, it appeared to be equally low when the firstborn child was either male or female. We also rejected our hypothesis that girls would be less likely to receive high-protein solid foods, such as meat, eggs, or fish. The only food items which differed were fresh milk and breast milk. Finally, we found evidence supporting our hypothesis that the magnitude of the observed gap in breastfeeding was attributable for a substantial portion of the inequalities in male and female child survival in India.

These findings have important implications for maternal and child health practices in India. First, the importance of continuing breastfeeding should be stressed during antenatal as well as postnatal care visits, as there is a female disadvantage in duration of breastfeeding that mainly emerges after the first year of life. Second, while the study has documented that young girls are disadvantaged, there is a need for future research to investigate the potential role of fertility preferences with regard to sons and daughters, which may help detect high-risk households. Third, as the evidence of nutritional disparities accounting for gendered mortality outcomes is only partial, there is scope to investigate potential gendered disparities in improving child health through access to clean drinking water, regular health care visits, and appropriate vaccination practices.

Further investigation is needed to understand the social and geographic determinants of these observed gendered nutritional disparities. There is also a need to better understand household risk factors, including the role of female autonomy given its known importance for maternal and child health [36], [37]. Importantly, future research is needed to investigate the possibility that differential healthcare access may further contribute to young girls’ disproportionately worse survival experience in India.
